# *Ophiorrhiza saxatilis* (Rubiaceae), a new species from Guizhou, China

**DOI:** 10.3897/phytokeys.278.197480

**Published:** 2026-07-24

**Authors:** Guang-Lin Li, Ming-Tai An, Xu Wu, Jiang-Hong Yu, Jian Qiu, Shao-Fei Liu, Cheng-jiang Tan

**Affiliations:** 1 College of Forestry, Guizhou University, Guiyang 550025, Guizhou, China College of Forestry, Guizhou University Guiyang China; 2 Key laboratory of Plant Resource Conservation and Germplasm innovation in Mountainous Region (Ministry of Education), College of Life Sciences/Institute of Agro-bioengineering, Guizhou University, Guiyang 550025, Guizhou, China Key laboratory of Plant Resource Conservation and Germplasm innovation in Mountainous Region (Ministry of Education), College of Life Sciences/Institute of Agro-bioengineering, Guizhou University Guiyang China; 3 Guizhou Maolan National Nature Reserve Administration, Libo 558400, Guizhou, China Guizhou Maolan National Nature Reserve Administration Libo China

**Keywords:** Karst, Morphology, New Taxon, *
Ophiorrhiza
*, Rubiaceae

## Abstract

*Ophiorrhiza saxatilis* M.T.An & G.L.Li, **sp. nov**., a new species of the genus *Ophiorrhiza* (Rubiaceae) discovered in the karst regions of Guizhou, China, and provides its taxonomic basis from a morphological perspective. The new species is morphologically similar to *Ophiorrhiza lignosa*, but differs from the latter by having an inflorescence in that its young branches are uniformly pubescent (vs. bifariously pubescent in *O. lignosa*); the abaxial leaf surface is pubescent (vs. puberulent only along the midrib and secondary veins in *O. lignosa*); the bracteoles are 5–6 mm long (vs. 1.5–3 mm long in *O. lignosa*); the petioles are 2–5.8 mm long (vs. 6–20 mm long in *O. lignosa*); the stamens are inserted at the base of the corolla tube (vs. inserted near the middle of the corolla tube in *O. lignosa*); the flowers are distylous (vs. monomorphic with pin flowers in *O. lignosa*), and the styles of long-styled flowers are 6–8 mm long (vs. 10–11 mm long in *O. lignosa*). These stable and significant morphological differences, combined with a comprehensive and detailed morphological description of the new species and a comparison of diagnostic characters with similar species, firmly support the recognition of *Ophiorrhiza saxatilis* as a distinct new species.

## Introduction

*Ophiorrhiza* L. is a member of tribe Ophiorrhizeae, subfamily Rubioideae, Rubiaceae (Bremer and Eriksson 2009), comprising 384 species worldwide (POWO 2026). *Ophiorrhiza* is a genus of considerable pharmacological importance, traditionally used as folk medicinal herbs in China and neighboring regions (Taher et al. 2020). It is a primary natural source of the potent anticancer monoterpene indole alkaloid camptothecin (CPT) (Wang et al. 2023), and *Ophiorrhiza pumila* serves as a key model plant for studying CPT biosynthesis and regulation (Shi et al. 2022). The genus is mainly distributed across tropical and subtropical Asia and the Pacific region, with New Guinea and Southeast Asia as its primary centers of diversity (Hareesh and Sabu 2018; Wu et al. 2019). China represents one of the most species-rich regions for this genus, with 85 species currently recorded (Biodiversity Committee of the Chinese Academy of Sciences 2025), most of which are distributed in southern and southwestern China (Lo 1999; Wu et al. 2018). In recent years, several new species of this genus have also been discovered in China (Yang et al. 2018; Liu et al. 2024; Zhan et al. 2024; Wang et al. 2026), highlighting this region as a key hotspot for undiscovered *Ophiorrhiza* diversity.

Located in Libo, Qiannan Buyi and Miao Autonomous Prefecture, Guizhou Province, and bordering Guangxi, the Maolan National Nature Reserve is a core area of the South China Karst World Natural Heritage site (Linghu and Ran 2009). Despite high rock exposure, shallow and scattered soil, and extremely poor surface water retention, the reserve preserves the largest and most pristine karst forest at the same latitude globally, harboring a unique ecosystem and rich biodiversity (Lang and Long 2012). In March 2025, during a field survey in this reserve, we discovered a subshrub with white flowers. Based on its leaf arrangement and floral morphology, we preliminarily identified it as a species of *Ophiorrhiza*. In May of the same year, during a follow-up survey in the Maolan National Nature Reserve, we observed fruit specimens of this species. Through detailed morphological comparison and study, we confirmed that it represents an undescribed new species of *Ophiorrhiza*.

## Materials and methods

### Morphological characteristics

In this study, flowers, leaf blades, petioles and young branchlets were selected as core morphological characters for systematic observation and data recording. All morphological measurements were obtained from 18 mature flowering individuals within the type population. For each individual, 10 fully expanded mature leaves were randomly sampled for measurement, and the morphological dimensions of vegetative and reproductive organs (including petioles, bracts and styles) were simultaneously determined and documented. Detailed morphological observations and trait measurements were first conducted on living plants in their *in situ* natural habitats. Supplementary morphological descriptions and calibration of measurement data were then performed on the collected specimens in the laboratory. Based on authoritative taxonomic monographs such as *Flora Reipublicae Popularis Sinicae* and *Flora of China*, we systematically sorted out the morphological characteristics of all published *Ophiorrhiza* species and identified the closely related taxa of the target group. The core diagnostic characters between the two taxa were clarified by comparing key traits against the type specimens of the closely related species. Furthermore, combined with herbarium specimen records from the Chinese Virtual Herbarium (https://www.cvh.ac.cn) and the National Specimen Information Infrastructure (http://www.nsii.org.cn), additional comparative verification was carried out between our newly collected materials and the closely related species.

On this basis, we accessed voucher specimens deposited in the Herbarium of Arnold Arboretum, Harvard University (A), the Herbarium of New York Botanical Garden (NY), the Herbarium of Kunming Institute of Botany, Chinese Academy of Sciences (KUN), and the Herbarium of South China Botanical Garden, Chinese Academy of Sciences (IBSC), and conducted systematic morphological observations and quantitative trait measurements on the new species and its morphologically close relative *O. lignosa*. Well-preserved specimens with intact morphological traits were selected for statistical analysis, including 6 specimens of *O. lignosa* (containing its holotype specimen) and 6 specimens of the new species (containing its holotype specimen). Nine morphological characters with stable and significant interspecific differences were screened for trait variation statistics and Principal Component Analysis (PCA) (Table 2). Based on Gower’s general similarity coefficient, trait variation analysis and Principal Coordinates Analysis (PCoA) were implemented using the vegan (Dixon 2003) and ggplot2 (Wickham 2016) packages in R.

**Table 1.** Morphological characteristics observed and measured in *O. saxatilis* and *O. lignosa*.

	**State**	**Type**	**Coding**
1	leaf length (cm)	quantitative	
2	leaf width (cm)	quantitative	
3	petiole length (mm)	quantitative	
4	internode length (cm)	quantitative	
5	lateral vein number	quantitative	
6	leaf shape	qualitative	lanceolate(0), linear (1)
7	leaf texture	qualitative	papery(0), thinly leathery (1)
8	stem hair	qualitative	bifariously pubescent (0), uniformly pubescent (1)
9	leaf hair	qualitative	presence (0), absence (1)

**Table 2.** Morphological comparison between *Ophiorrhiza saxatilis* and *O. lignosa*.

	***O. saxatilis***	***O. lignosa***
leaf texture	thinly leathery	papery
leaf shape	linear, apex acute	lanceolate, acuminate
leaf size	2–8 cm long, 0.5–1.2 cm wide	5–11 cm long, 1–2 cm wide
abaxial leaf surface	pubescent	abaxially puberulent along midrib and secondary veins
abaxial midrib	purple	green
Petiole	2–5.8 mm long	6–20 mm long
young branches	uniformly pubescent	bifariously pubescent
corolla color	white	purple
stamen insertion position	inserted at base of corolla tube	inserted near middle of corolla tube
Style	long-styled flowers 6–8 mm, short-styled flowers 2–4 mm	long-styled flowers 10–11 mm, short-styled flowers absent
Bracteoles	5–6 mm long	1.5–3 mm long

Comprehensive analysis of the closely related taxa was performed by integrating field survey records, *in situ* photographs and herbarium specimen information, and the core diagnostic characters of the new species were finally determined. All voucher specimens of the new species are deposited in the Herbarium of College of Forestry, Guizhou University (**GZAC**).

## Results

### Morphological characteristics

We systematically observed and analyzed morphological characters from a total of 12 specimens of *O. saxatilis* and *O. lignosa*. The two species can be reliably distinguished by a set of diagnostic traits, including leaf size and texture, petiole length, pubescence pattern of young branches, bracteole length, style morph type, and stamen insertion position (Fig. 5, Table 2). Principal coordinate analysis (PCoA) revealed a clear morphological separation between the two taxa (Fig. 5J).

### Taxonomic treatment

#### Ophiorrhiza saxatilis

Taxon classificationPlantaeGentianalesRubiaceae

M.T.An & G.L.Li sp. nov.

7D1408EF-FEA5-5595-A78F-665094F84CDF

urn:lsid:ipni.org:names:77393145-1

Figs 1, 2, 3, 4

##### Type.

China, • Guizhou Province, Libo County, Maolan National Nature Reserve; 25°17'N, 107°55'E (Fig. 3), elevation 960 m; 8 May 2025; *Ming-tai An, Guang-lin Li, Jiang-hong Yu, GZAC-LGL-250429* (holotype: GZAC-LGL-1; isotype: GZAC-LGL-2).

**Figure 1.**
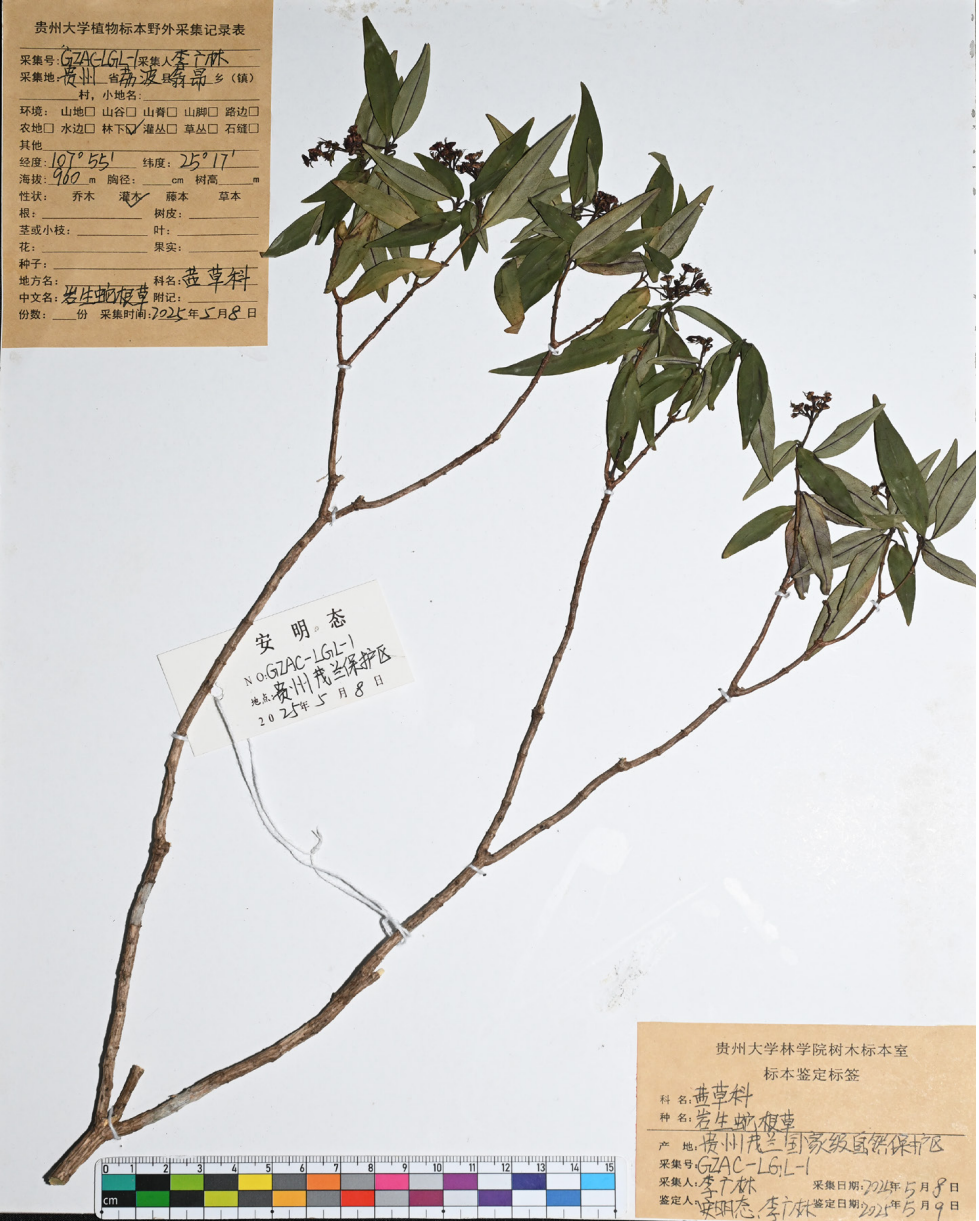
Holotype of *Ophiorrhiza saxatilis*.

**Figure 2.**
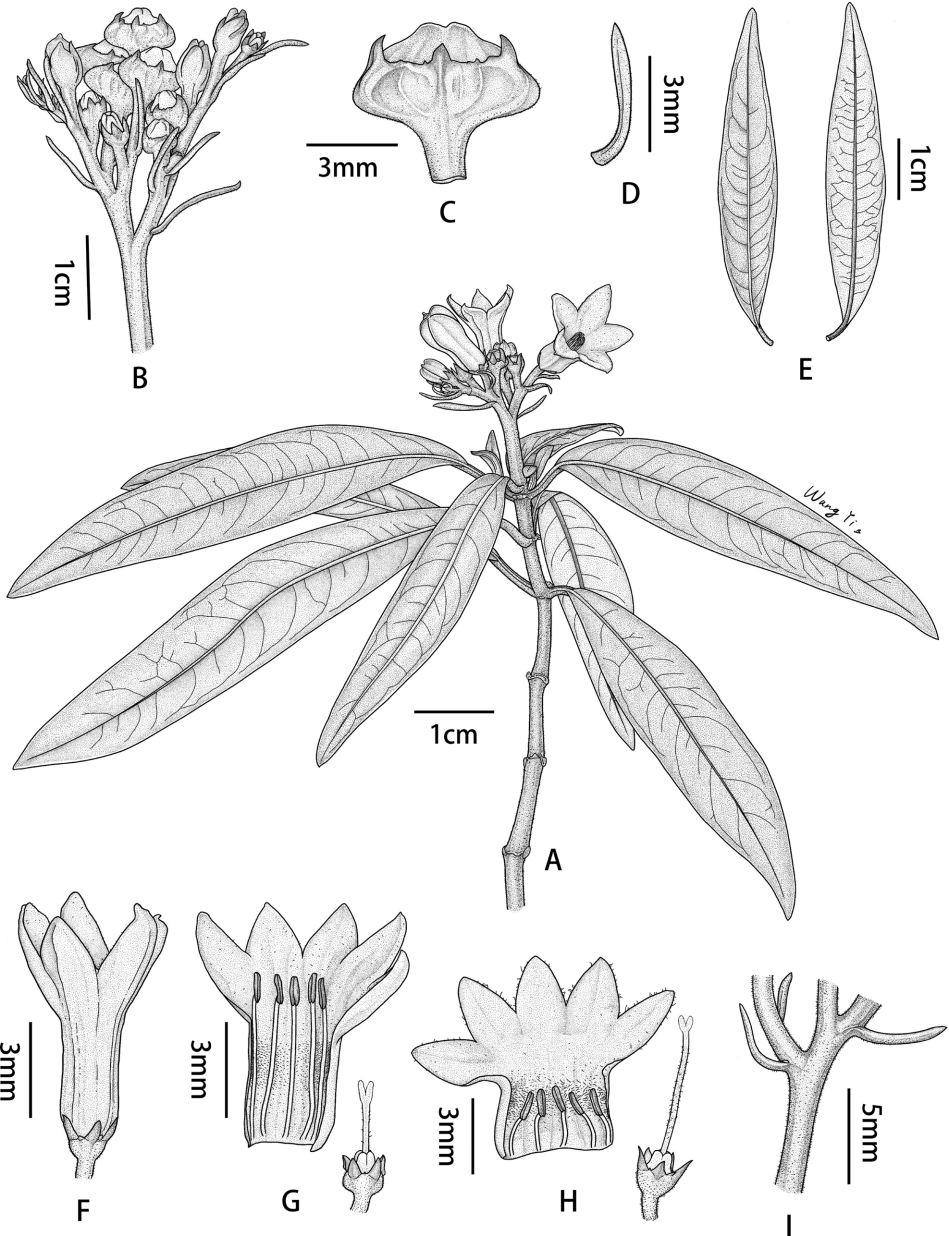
*Ophiorrhiza saxatilis*. **A**. Flowering branch; **B**. Infructescence; **C**. Fruit; **D**. Bracteoles; **E**. Adaxial (left) and abaxial (right) leaf surface; **F**. Lateral view of flower; **G**. Longitudinally dissected short-styled flower; **H**. Longitudinally dissected long-styled flower; **I**. Branchlets. Drawn from the holotype by Yi Wang.

**Figure 3.**
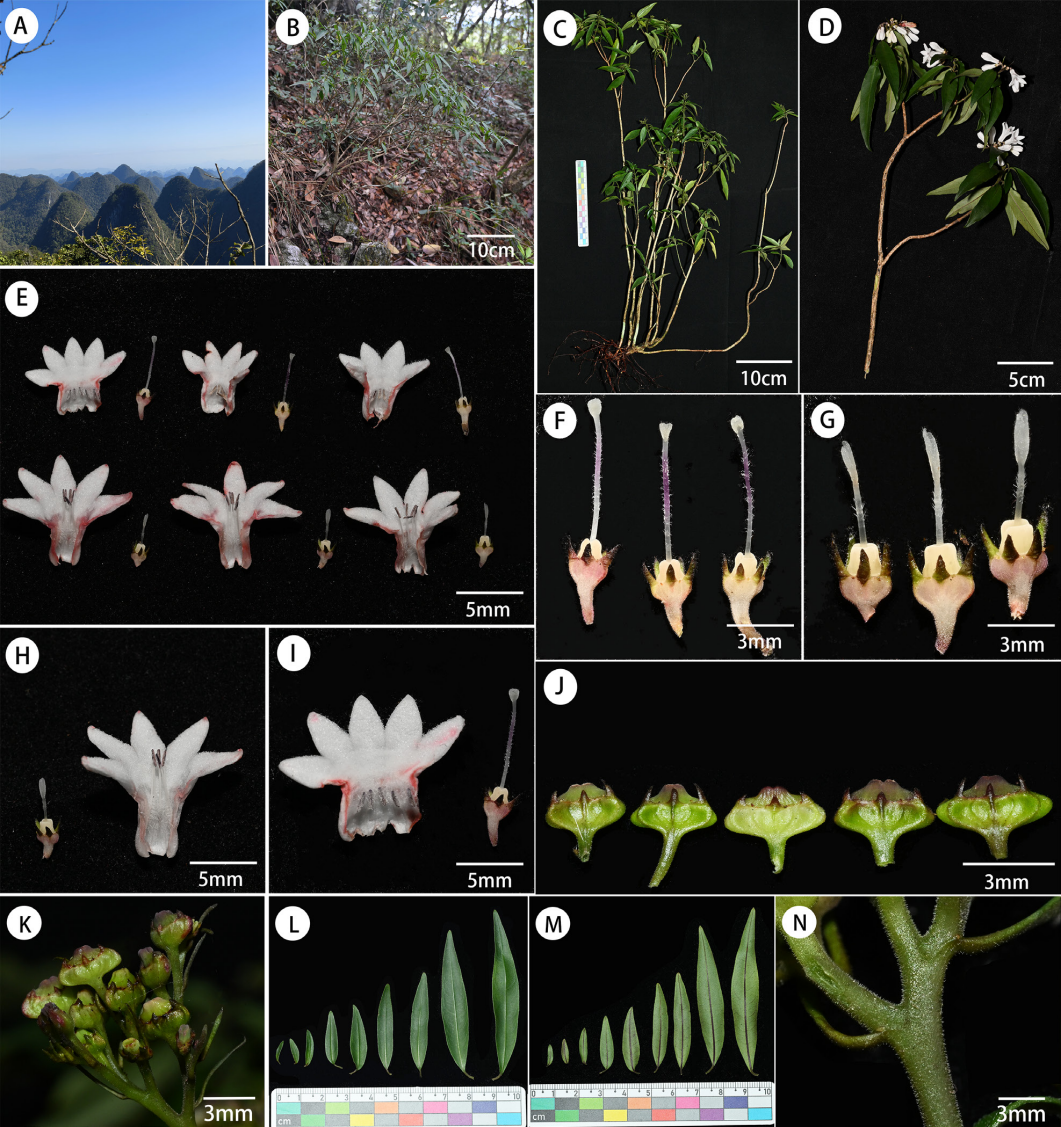
*Ophiorrhiza saxatilis*. **A, B**. Habitat; **C**. Plant; **D**. Flowering branch; **E**. Dissected flowers: long-styled flower (above) and short-styled flower (below); **F**. Style of long-styled flower; **G**. Style of short-styled flower; **H**. Longitudinally dissected short-styled flower; **I**. Longitudinally dissected long-styled flower; **J**. Fruit; **K**. Infructescence; **L**. Adaxial leaf surface; **M**. Abaxial leaf surface; **N**. Branchlets.

**Figure 4.**
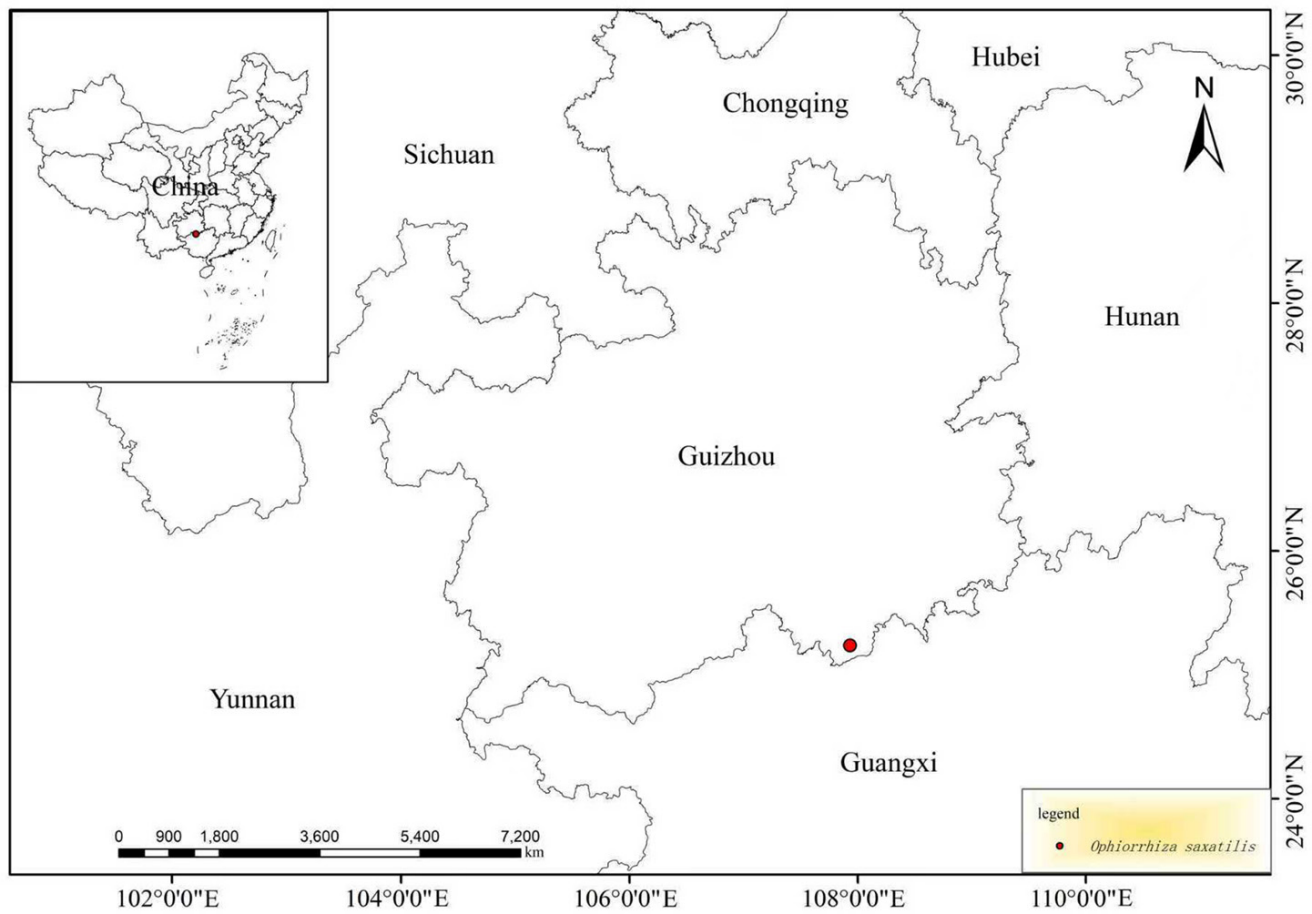
Distribution of *Ophiorrhiza saxatilis*.

**Figure 5.**
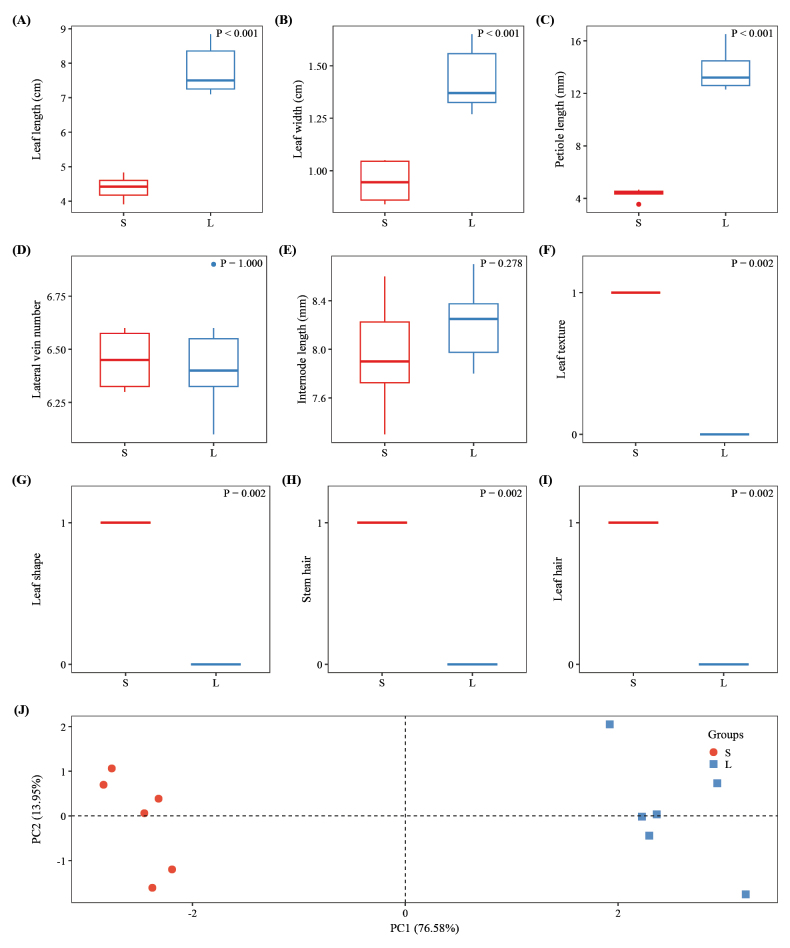
Variation in 9 morphological characters (**A–I**) and principal coordinate analysis (**J**) of *Ophiorrhiza saxatilis* and *O. lignosa*. Specimens information for *O lignosa*: F. Kingdon-Ward 252 Holotype (**A**), F. Kingdon-Ward 252 Isotype (NY), F. Kingdom-Ward 352 Isotype (NY), En-de Liu, Fa-zhi Shangguan, Zhi-dan Wei, Xin-xin Zhu, Zeng-peng Sun, Bo Xiao 6852 (KUN), Zhi-dan Wei, Fa-zhi Shangguan, Xin-xin Zhu, Bo Xiao, Geng-shen Wang, Jun Wang, LiuED8753 (KUN), Qi-wu Wang 86399 (IBSC). Specimens’ information for *O. saxatilis*: Ming-tai An, Guang-lin Li, Jiang-hong Yu GZAC-LGL-1 (Holotype), GZAC-LGL-2 (Isotype) GZAC-LGL-3, GZAC-LGL-4, GZAC-LGL-5, GZAC-LGL-6 (GZAC).

##### Diagnosis.

*Ophiorrhiza saxatilis* is morphologically similar to *O. lignosa* Merr., but can be distinguished by several key characteristics (Table 2): uniformly pubescent young branches (vs. bifariously pubescent); white corolla lobes with ridges (vs. purple, without ridges); stamens inserted at the base of the corolla tube (vs. inserted near the middle of the corolla tube); flowers distylous (vs. monomorphic, with pin flowers); long-styled flowers 6–8 mm long (vs. 10–11 mm long); purple midrib on the abaxial leaf surface (vs. green); abaxial leaf surface pubescent (vs. puberulent only on the midrib and secondary veins); bracteoles 5–6 mm long (vs. 1.5–3 mm long); and petioles 2–5.8 mm long (vs. 6–20 mm long).

##### Etymology.

The specific epithet is derived from the habitat of the new species, which occurs in a typical karst limestone environment. It mainly grows in harsh habitats such as rock crevices and stony slopes.

##### Local name.

Simplified Chinese: 岩生蛇根草; Chinese Pinyin: yán shēng shé gēn cǎo.

##### Description.

Subshrubs, to 1 m tall; older branches gray, terete, woody; young branches pubescent, drying obscurely 4–angled. Leaves thinly leathery, linear or narrowly oblong, 2–8 cm × 0.5–1.2 cm, margins entire, drying pale green and sparsely pubescent adaxially, purple-red and pubescent abaxially; midrib flat adaxially, prominent and purple abaxially; secondary veins slender, 7–9 pairs; tertiary venation indistinct; petiole 2–5.8 mm, puberulent; stipules caducous, not seen. Inflorescences relatively small, terminal, many-flowered; peduncle 1–2 cm, pubescent; axes helicoid, up to 1 cm, pubescent; pedicels slender, ca. 0.5–0.8 mm; bracteoles linear, 5–6 mm, pubescent. Calyx hispidulous; hypanthium compressed-turbinate, 5–ribbed, ca. 1 mm × 2 mm; lobes 5, ovate-triangular, 0.4–0.7 mm, glands often present in sinuses. Flowers distylous, white, drying purple; corolla tubular, glabrous outside, with a white villous ring at the middle inside, throat pubescent; tube 4–5 mm; lobes broadly ovate or oblong-ovate, ca. 4 mm, distinctly keeled. Short-styled flowers: stamens inserted at the base of the corolla tube, filaments elongated, exserted; anthers ca. 1.4 mm; style 2–4 mm, hispid; stigma oblong-ellipsoid, bilobed, ca. 1.6 mm. Long-styled flowers: style slightly exserted, 6–8 mm, villous; stigma shallowly bilobed, suborbicular, 0.8–1 mm. Capsules mitriform, 6–8 mm wide, 2.5–3 mm tall, puberulent. Seeds angular.

##### Phenology.

Flowering from February to April, fruiting from April to June.

##### Distribution and habitat.

*Ophiorrhiza saxatilis* is currently known only from southwestern China (Libo County, Guizhou Province). This species grows in forests on karst hills at an altitude of approximately 960 m, in a habitat characterized by high rock exposure, poor and barren soil, and low soil water retention capacity.

##### Conservation status.

During field surveys, we discovered a wild population of *O. saxatilis*. This species mainly inhabits the middle to upper parts of karst hills. In the vicinity of the surveyed area, we also observed scattered individuals of this species, with a total of approximately 150 plants. As our current investigation into the conservation status and threatening factors of *O. saxatilis* is not comprehensive enough to provide detailed information on the population size and distribution, we propose that *O. saxatilis* be categorized as “Data Deficient” (IUCN 2024).

## Discussion

This paper reports *Ophiorrhiza saxatilis*, a new species of the genus *Ophiorrhiza* discovered in the limestone mountains of southwestern China. This discovery enriches our understanding of the taxonomic diversity of the genus, particularly in the karst regions of China, which are considered among the most species-rich areas for *Ophiorrhiza*. Plants of *Ophiorrhiza* are mostly herbs, creeping or erect, commonly found in large patches along gully edges or in shallow silt deposits under dense forests, favoring humid environments (Lo 1990). Taxa with distinctly woody stems are relatively rare within the genus, and this woody characteristic holds particular adaptive significance, likely reflecting an evolutionary outcome of long-term adaptation to specific karst habitats. Morphometric analysis between *O. saxatilis* and *O. lignosa* (Fig. 5) revealed that their primary diagnostic differences are reflected in morphological traits including branchlet indumentum, petiole length, leaf characters and flower type. A detailed morphological comparison between the two species is presented in Table 2. These characters collectively form a unique suite of morphological traits, with stable differences, clearly distinguishing *O. saxatilis* from *O. lignosa*. This finding underscores the importance of meticulous morphological examination within the highly diverse genus *Ophiorrhiza* and highlights the rich, yet still incompletely documented, biodiversity of karst forests in southern China and adjacent regions.

### Additional specimen examined

*Ophiorrhiza lignosa* Merr.— Myanmar (Burma). Hkam-Kawng: Ngawchang Valley, roadside banks in forest, alt. 4000 ft (ca. 1219 m), Mar. 1, 1939, F. Kingdon-Ward 252 (A, NY); Hkam-Kawng: Ngawchang Valley, roadside banks in forest, undershrub, alt. 4000 ft (ca. 1219 m), Mar. 1, 1939, F. Kingdon-Ward 352 (NY).— China. Yunnan: Tiechang Township, Malipo County, Wenshan Zhuang and Miao Autonomous Prefecture, under forest, 23.424444°N, 105.068359°E, alt. 1477 m, May 18, 2017, En-de Liu, Fa-zhi Shangguan, Zhi-dan Wei, Xin-xin Zhu, Zeng-peng Sun, Bo Xiao 6852 (KUN); Bajiao Ping Village, Donggan Township, Malipo County, Wenshan Zhuang and Miao Autonomous Prefecture, evergreen broad-leaved forest, 22°59'3.48"N, 104°50'19.08"E, alt. 1160 m, Mar. 25, 2018, Zhi-dan Wei, Fa-zhi Shangguan, Xin-xin Zhu, Bo Xiao, Geng-shen Wang, Jun Wang, LiuED8753 (KUN); Malipo, border of dense woods, alt. 1100 m, Jan. 21, 1940, Qi-wu Wang 86399 (IBSC).

## Supplementary Material

XML Treatment for Ophiorrhiza saxatilis

## Additional information

### Artificial Intelligence (AI) use

The authors accept full responsibility for the content of the manuscript, including the disclosure of any use of AI.

No AI tools were used in the preparation of this manuscript.

### Author ORCIDs

G.-L. Li https://orcid.org/0009-0009-5324-4643

M.-T. An https://orcid.org/0000-0003-3886-0287

X. Wu https://orcid.org/0009-0004-7935-6057

J.-H. Yu https://orcid.org/0000-0003-1765-8557

### Data availability

All of the data that support the findings of this study are available in the main text or Supplementary Information.

## Supplementary materials

Supplementary material 1 Raw individual morphological data of *O. saxatilis* and *O. lignosa* used for interspecific comparison and principal component analysis Data type xlsx https://binary.pensoft.net/file/1725083This dataset is made available under the Open Database License (http://opendatacommons.org/licenses/odbl/1.0/). The Open Database License (ODbL) is a license agreement intended to allow users to freely share, modify, and use this Dataset while maintaining this same freedom for others, provided that the original source and author(s) are credited. Guang-Lin Li, Ming-Tai An, Xu Wu, Jiang-Hong Yu, Jian Qiu, Shao-Fei Liu, Cheng-jiang Tan
